# Different sex ratios of children born to Indian and Pakistani immigrants in Norway

**DOI:** 10.1186/1471-2393-10-40

**Published:** 2010-08-03

**Authors:** Narpinder Singh, Are Hugo Pripp, Torkel Brekke, Babill Stray-Pedersen

**Affiliations:** 1 Division of Obstetrics and Gynecology, Rikshospitalet University Hospital, Oslo, Norway; 2 Department of Research Services, Rikshospitalet University Hospital, Oslo, Norway; 3 Section of Culture and Oriental studies, University of Oslo, Oslo, Norway; 4 Faculty of Medicine, University of Oslo, Oslo, Norway

## Abstract

**Background:**

A low female-to-male ratio has been observed in different Asian countries, but this phenomenon has not been well studied among immigrants living in Western societies. In this study, we investigated whether a low female-to-male ratio exists among Indian and Pakistani immigrants living in Norway. In particular, we investigated whether the determination of sex via ultrasound examination, a common obstetric procedure that has been used in Norway since the early 1980 s, has influenced the female-to-male ratio among children born to parents of Indian or Pakistani origin.

**Methods:**

We performed a retrospective cohort study of live births in mothers of Indian (n = 1597) and Pakistani (n = 5617) origin. Data were obtained from "Statistics Norway" and the female-to-male (F/M) sex ratio was evaluated among 21,325 children born, in increasing birth order, during three stratified periods (i.e., 1969-1986, 1987-1996, and 1997-2005).

**Results:**

A significant low female-to-male sex ratio was observed among children in the third and fourth birth order (sex ratio 65; 95% CI 51-80) from mothers of Indian origin who gave birth after 1987. Sex ratios did not deviate from the expected natural variation in the Indian cohort from 1969 to 1986, and remained stable in the Pakistani cohort during the entire study period. However, the female-to-male sex ratio seemed less skewed in recent years (i.e., 1997-2005).

**Conclusion:**

Significant differences were observed in the sex ratio of children born to mothers of Indian origin compared with children born to mothers of Pakistani origin. A skewed number of female births among higher birth orders (i.e., third or later) may partly reflect an increase in sex-selective abortion among mothers of Indian origin, although the numbers are too small to draw firm conclusions. Further research is needed to explain the observed differences in the female-to-male ratio among members of these ethnic groups who reside in Norway.

## Background

Female infanticide is a method of sexual selection that is practiced in areas of the world where male children are valued over female children. Recent findings based on data from the United Kingdom's national register revealed a low female-to-male ratio at birth to Indian-born mothers living in England and Wales. This skewed sex ratio was observed specifically among higher birth orders [1]. Tendency toward a skewed sex ratio has also been found among families of Asian ethnicity living in Quebec [2].

Worldwide, the annual sex ratio is approximately 95 females to 100 males, and may fluctuate somewhat among different races [3]. Many biological factors influence the normal sex ratio [4-9]. However, the pronounced decrease in female births in Asian countries such as India [10], China [11] and South Korea [12] is too extreme to result solely from biological variation. The skewed sex ratio has been attributed to an increase in sex-selective abortion, in addition to female infanticide [13]. Moreover, skewed sex ratios are more common among higher births orders in families without male offspring [10]. Therefore, sex-selective induced abortion may explain the findings observed in higher birth orders.

Many immigrants from Asian regions with skewed sex ratios belong to cultures with a high preference for sons rather than daughters. However, sex-selective induced abortion is strongly opposed in Western culture and is illegal except for medical reasons. The skewed sex ratios among immigrant groups in UK have raised concerns about disclosing the sex of the fetus during an ultrasound examination. The abortions of female fetuses raise doubts about the adherence of Asian immigrants to the norms of Western society [1].

In the 1970 s, immigrants began traveling to Norway from India and Pakistan. Norway is currently home to 4.8 million people, including about 7,000 immigrants from India and about 68,000 immigrants from Pakistan, according to 2005 estimates. Most immigrants are from the province of Punjab [14], which was divided between India and Pakistan during the partition in 1947. The two groups shared a geographical space in northwest India until the creation of India and Pakistan, and are fairly similar in terms of language and culture. However, Indians from Punjab are predominantly Sikh and Hindu, whereas most Pakistani immigrants are Muslim. These immigrants belong to societies with a high degree of boy preference and there is anecdotal evidence of sex-selective abortion or prenatal male selection within the Indian community in Norway. However, no studies, to our knowledge, have addressed the prevalence of these practices among members of these groups in Scandinavia.

Thus, the aim of the present study was to determine whether the low female-to-male ratio common in North-West Indian cultures also exists among families from the Indian subcontinent who live in Norway.

## Methods

### Data collection

We performed a retrospective cohort study using data from mothers of Indian (n = 1,597) or Pakistani (n = 5,617) origin who were registered as immigrants in Norway. Annual data were obtained from 'Statistics Norway' for 21,325 live births between 1969 and 2005. The dataset included the infants' sex, the mothers' nationality, and the infants' birth order. The study was approved by the Data Inspectorate at Rikshospitalet, Oslo University Hospital, Norway.

### Statistical analysis

Sex ratios and 95% confidence intervals were defined as described previously by Jha et al. [8] as the number of female births per 100 male births [Pf/(1 - Pf) * 100], where Pf is the proportion of female to total births (N). Confidence intervals (95%) were derived based on an asymptotic normal distribution with a variance of Pf/[N * (1 - Pf) ^3^]. Plots of sex ratio for the first and last (if the mother had more than one birth) birth order against the birth year were constructed for the Indian and Pakistani cohorts. We performed a regression analysis of the trend in the annual sex ratio at birth between 1969 and 2005. Linear and quadratic regression models were fitted to the data, and the model with best fit based on adjusted R^2 ^values was selected. All statistical analyses were performed using SPSS version 16.0 (SPSS Inc., Chicago, IL).).

## Results

### Indian immigrants

From 1969 to 2005, a total of 1,597 mothers of Indian origin gave birth in Norway to 3,525 children, and 19% of these children were in the third birth order or higher. From 1969 to 1986, the sex ratios were 108 (95% CI 75-141) and 93 (95% CI 43-142) in the third and fourth birth orders, respectively (Table 1). These results are consistent with the expected biological sex ratio (i.e., 95 females per 100 males). During the period from 1987 to 1996, sex ratios of 62 and 36 were estimated for the third and fourth birth orders, respectively. During the final study period from 1997 to 2005, relatively low sex ratios of 69 and 47 were estimated for the third and fourth birth orders, respectively.

**Table 1:** Female and male births and sex ratios among Indian and Pakistani populations living in Norway

Time period (year)	Birth order of child	Indian			Pakistani		
		Female	Male	Sex ratio (95% CI)	Female	Male	Sex ratio (95% CI)
**1969 - 1986**	1	259	297	87 (73 - 102)	992	1025	97 (88 - 105)
	
	2	197	207	95 (77 - 114)	845	904	93 (85 - 102)
	
	3	85	79	108 (75 - 141)	598	671	89 (79 - 99)
	
	4	26	28	93 (43 - 142)	357	370	96 (82 - 111)

							

**1987 - 1996**	1	283	250	113 (94 - 132)	726	780	93 (84 - 102)
	
	2	208	204	102 (82 - 122)	644	642	100 (89 - 111)
	
	3	64	103	62 (43 - 82)	565	546	103 (91 - 116)
	
	4	12	33	36 (12 - 60)	425	427	100 (86 - 113)

							

**1997 - 2005**	1	273	235	116 (96 - 136)	1002	1092	92 (84 - 100)
	
	2	202	237	85 (69 - 101)	836	818	102 (92 - 112)
	
	3	68	99	69 (47 - 90)	578	575	101 (89 - 112)
	
	4	8	17	47 (8 - 87)	289	324	89 (75 - 103)

Female and male births and respective sex ratios (i.e., female/male) with 95% confidence intervals for the study periods between 1969 and 1986, between 1987 and 1996 and between 1997 and 2005 among the Indian and Pakistani populations living in Norway.

### Pakistani immigrants

During the period from 1969 to 2005, 5,617 mothers of Pakistani origin gave birth in Norway to 17,800 children, and 42% of the children were in the third birth order or higher. From 1969 to 1986, the sex ratios were 89 (95% CI 79-99) and 96 (95% CI 82-111) in the third and fourth birth orders, respectively (Table 1). In the period 1987-1996, sex ratios of 103 and 100 were estimated for third and fourth birth orders, respectively. During the final study period from 1997 to 2005, sex ratios of 101 and 89 were estimated for the third and higher birth orders, respectively.

### Regression analysis of the annual trend

**Figure 1:**
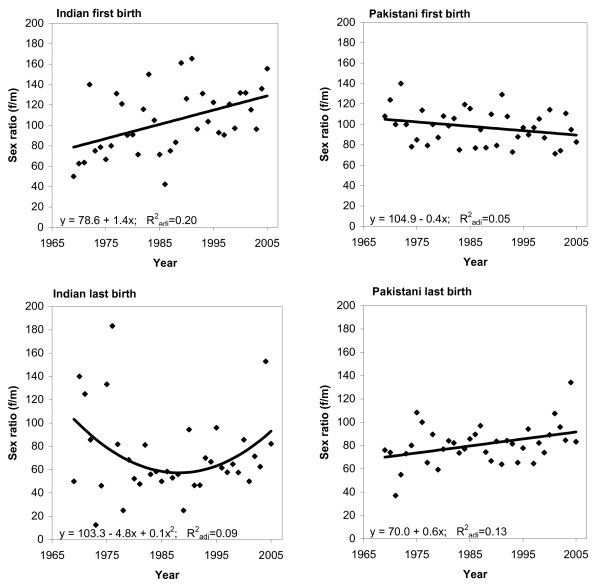
**Estimated female-to-male sex ratios from 1970 to 2005 for children born to women of Indian and Pakistani origin in the first and last (i.e., among mothers with two or more children) birth orders**.

Linear and quadratic regression models were used to evaluate the annual trends in sex ratios for the first and last birth order in the Indian and Pakistani cohort (Figure 1). Our results show that for the first birth order, the female-to-male sex ratio increased in the Indian cohort and decreased in the Pakistani cohort. The sex ratio increased steadily in the Pakistani cohort for the last birth order. Conversely, the ratio of female births decreased in the Indian cohort from the 1970 s to the early 1990 s but has since increased.

## Discussion

Our findings reveal that the female-to-male ratio of children born to Indian immigrants who reside in Norway is skewed among the higher birth orders, and has become increasingly distorted since the mid-1980 s. This trend corresponds to the availability of ultrasound techniques capable of determining the sex of the fetus during the prenatal period [15]. Thus, the distorted ratio observed during the two periods may reflect increases in sex-selective abortion of higher birth order fetuses. More girls than boys compose the first birth order in the Indian immigrant group, and fewer girls than boys compose the higher birth order. These data indicate that most families secure at least one male child. Although the number of births among the Indian population in Norway is too low to provide robust statistical evidence for a skewed sex ratio, the decrease in female births at higher birth orders shows a tendency towards sex selection.

Studies have indicated that educated mothers in India have the lowest female-to-male ratio [10]. The odds of producing a male offspring in India increase with the mother's income and education [16]. Previous studies have shown that the female-to-male ratio in Pakistan is not as skewed as it is in India, and that sex-selective abortion may not be as prevalent in Pakistan as in India [17]. In Norway, the two immigrant groups differ significantly in terms of integration into the Norwegian society, educational status and employment status. Indian immigrants belong to one of the most resourceful immigrant groups in Norway [18]. Economically sound and educated Indian women often produce a smaller family, both in their native country and in Western countries. The number of children born to Indian-born women in England and Wales declined from 4.3 in 1971 to 2.3 in 2001 [1]. Our data also indicate that families are smaller among Indian populations than among Pakistani populations living in Norway. The differences in family size and socio-economic variables among Indian and Pakistani populations in Norway suggest that Indian mothers opt for sex-selective abortion of higher birth orders, although further studies are needed to confirm this practice.

Cultural practices may influence a family's decision to undergo sex-selective abortion. Although male preference, and subsequent discrimination against female children, is widely documented in India and Pakistan owing to factors such as kinship, dowry, employment, education and religion [19], unbiased sex ratios and the use of sex-selective abortion differs between Indians and Pakistanis. A review of abortion practice in Islamic countries concluded that there is no single Islamic or Muslim position on abortion. There are considerable differences in state policies and decisions about the termination of pregnancy. Therefore, there is no absolute link between state religion and abortion prevalence [20]. No significant differences in adjusted sex ratios (e.g., male birth following female birth) were found among different religious groups in India [10]. It is likely that the selective sex practices of the two populations in our study might reflect subconscious cultural practices rather than religion. Furthermore, India and Pakistan have different laws regarding abortion. Pakistan has much stricter abortion legislation, and abortions are prohibited before the fetal organs have developed, except when performed to save the life of the woman or to provide necessary treatment. Termination of pregnancy after the organs have formed is prohibited regardless of the circumstances. Conversely, India and China have more liberal abortion laws and sex-selective abortion is widely documented [21]. The cultural and legislative factors of their native region might influence the prevalence of sex-selective abortion among immigrants to Western countries.

Sex-selective abortion remains a large problem due to the low status of women and the preference for male offspring among South-Asian individuals [21]. Previous studies have suggested that the methods of sexual selection are changing. Nowadays, technologies enable the implantation of embryos of a specific sex, and prenatal selection of male embryos has become more common [22]. Sex-selective abortion to secure a male offspring conflicts with Norwegian ethics and social norms, and the negative consequences have been discussed in leading national medical journals [23]. As shown by a study conducted in England and Wales, Indian-born immigrant mothers have a low female-to-male ratio in higher birth orders, but that pattern is not observed in mothers of Indian descent who are born in England and Wales [1]. Given the small number of Indian women in Norway, we were unable to assess the sex ratios of infants born to Norwegian-born mothers of Indian origin.

In summary, our findings reveal a low female-to-male ratio among higher birth orders among the Indian Diaspora in Norway. A similar trend has been reported among Indian and Asian populations in the UK and Canada [1,2]. Strengthening the social, economical and educational rights of women may eventually reduce sex-selective behavior in Asian regions and among immigrant families in Western countries.

## Conclusion

Our findings indicate that the female-to-male ratio of higher birth order children seems to have declined among Indian immigrants, but not among Pakistani immigrants, after the introduction of ultrasound scanning technology in Norway in 1987. Lower proportions of female births than expected were not found in the pre-ultrasound era. This imbalance could reflect the selective abortion of female fetuses due to prenatal sex determination by ultrasound. Further research is needed to reveal how religious, ethnic and socioeconomic factors contribute to the occurrence and norms of sex-selective abortion.

## Competing interests

The authors declare that they have no competing interests.

## Authors' contributions

The study was initiated by NS, who helped collect data, design and coordinate the study, and draft the manuscript. The statistical analyses were performed by AHP. TB participated in data collection. BSP contributed to the study design and helped plan, draft and finalize the manuscript. All authors read and approved the final amnauscript.

## Pre-publication history

The pre-publication history for this paper can be accessed here:

http://www.biomedcentral.com/1471-2393/10/40/prepub
